# Prophylactic femoral neck fixation in distal femur fractures: evaluating protection against ipsilateral hip fracture

**DOI:** 10.1007/s00590-026-04814-x

**Published:** 2026-08-21

**Authors:** Brian D. Rust, Daniel E. Pereira, David W. Barton, Mitchel R. Obey, Jenna-Leigh Wilson, Christopher M. McAndrew, Marschall B. Berkes

**Affiliations:** 1 Department of Orthopaedic Surgery, Washington University School of Medicine, Saint Louis, MO USA; 2 Department of Orthopaedics and Rehabilitation, Penn State College of Medicine, Hershey, PA USA

**Keywords:** Distal femur fracture, Lower extremity, Femoral neck, Hip fracture, Prophylactic fixation

## Abstract

**Purpose:**

Distal femur fractures occur in older, osteoporotic patients and carry a measurable risk of subsequent ipsilateral hip fracture. Prophylactic femoral neck screw placement is believed by many to mitigate this risk, but clinical evidence is limited. This study evaluates the association between prophylactic femoral neck fixation after distal femur fracture and future ipsilateral hip fracture.

**Methods:**

A retrospective cohort study was conducted at a single Level 1 tertiary trauma center between 2018 and 2024. Adult patients (≥ 18 years) with AO 33A/B/C fractures and Su type 1–3 periprosthetic fractures of the distal femur fracture treated with operative fixation were included. Patients with pathologic fractures, prior hip arthroplasty, or isolated antegrade intramedullary nailing were excluded. Treatment was classified by the presence or absence of ipsilateral prophylactic femoral neck screw fixation. The primary outcome of interest was the incidence of subsequent ipsilateral hip fracture.

**Results:**

Of 395 patients, 163 (41%) received prophylactic femoral neck fixation. The prophylactic screw (PS +) cohort included more female patients (81.0% vs. 68.5%, *p* = 0.006) and more periprosthetic fractures (65.0% vs. 35.8%, *p* < 0.001). Fixation constructs differed significantly, with nail-plate combinations predominating in the PS + group (69.1% vs 26.4%, *p* < 0.001). Six patients (1.5%) sustained an ipsilateral hip fracture. No difference was observed in the incidence of ipsilateral hip fracture by prophylactic femoral neck fixation (1.2% vs 1.7%, *p* = 0.691) or fracture-free survival (*p* = 0.802).

**Conclusion:**

Prophylactic femoral neck screw placement was not associated with a reduced rate of ipsilateral hip fracture after distal femur ORIF. Hip fractures occurred in both groups, suggesting that screw placement alone may not be independently protective.

## Introduction

Distal femur fractures are relatively common injuries (8.7/100,000 patients/year) that occur in older patients with osteoporosis as a mechanism of lower energy injuries [1]. As these injuries increase in incidence, particularly those around a total knee arthroplasty, so do their complications after surgery [2–4]. For example, ipsilateral femoral neck fractures occur following 1–7% of distal femur fractures [5–8]. Thus, optimization of this osteoporotic and frail population to avoid further osteoporotic fractures is warranted. Efforts to that end entail a multifaceted strategy that include nutritional supplementation, adequate protein intake, fall prevention, and pharmaceutical intervention against osteoporosis [9, 10].

Prophylactic screw protection of the axis and neck of the femur has been proposed as a potential strategy to prevent subsequent proximal femur fracture after fixation of distal femur fracture [11–13]. Most of the discourse surrounding its use has been in the context of femoral shaft fractures, and, until recently, there has been very limited data on employing femoral neck screws after distal femur fractures [14]. Therefore, it is not known definitively if screw protection of the femoral head and neck prevents proximal femur fracture after the fixation of distal femur fractures.

For that reason, the purpose of this study was to present a large case series of distal femur fracture patients treated with constructs that included prophylactic femoral neck fixation. Therefore, it is hypothesized that the addition of a prophylactic screw into the neck decreases femoral neck fracture rates after surgery.

## Materials and methods

### Study design and patient population

A retrospective review was conducted in adult patients treated for a distal femur fracture. Adult patients (age ≥ 50) underwent open reduction internal fixation (ORIF) at a single Level 1, tertiary referral trauma center between June 11, 2018, and December 31, 2024. Patients were identified in the institution’s trauma database using CPT codes (CPT 27506, 27511, 27513, and 27514) and secondarily verified with direct chart review and radiographic verification. All AO 33A/B/C native fractures (33A = extra articular, 33B = partial articular, 33C = complete articular) and Su 1/2/3 periprosthetic fractures (Su 1 = proximal to the knee prosthesis, Su 2 = staring at the border of the anterior flange, Su 3 = distal to the anterior flange) were included [15]. Exclusion criteria included patients with a pathologic fracture, patients with pre-existing hip arthroplasty, and patients who received an isolated antegrade intramedullary nail. Fixation constructs included retrograde intramedullary nail (rIMN), lateral locking plate (LLP), dual plate constructs (DPC), and the combination of both rIMN and lateral plating to create a nail-plate construct (NPC). Construct selection was determined according to the sole discretion of the primary surgeon. This study was submitted and accepted by the institutional review board for human research.

### Collected variables and reported outcomes

**Fig. 1:**
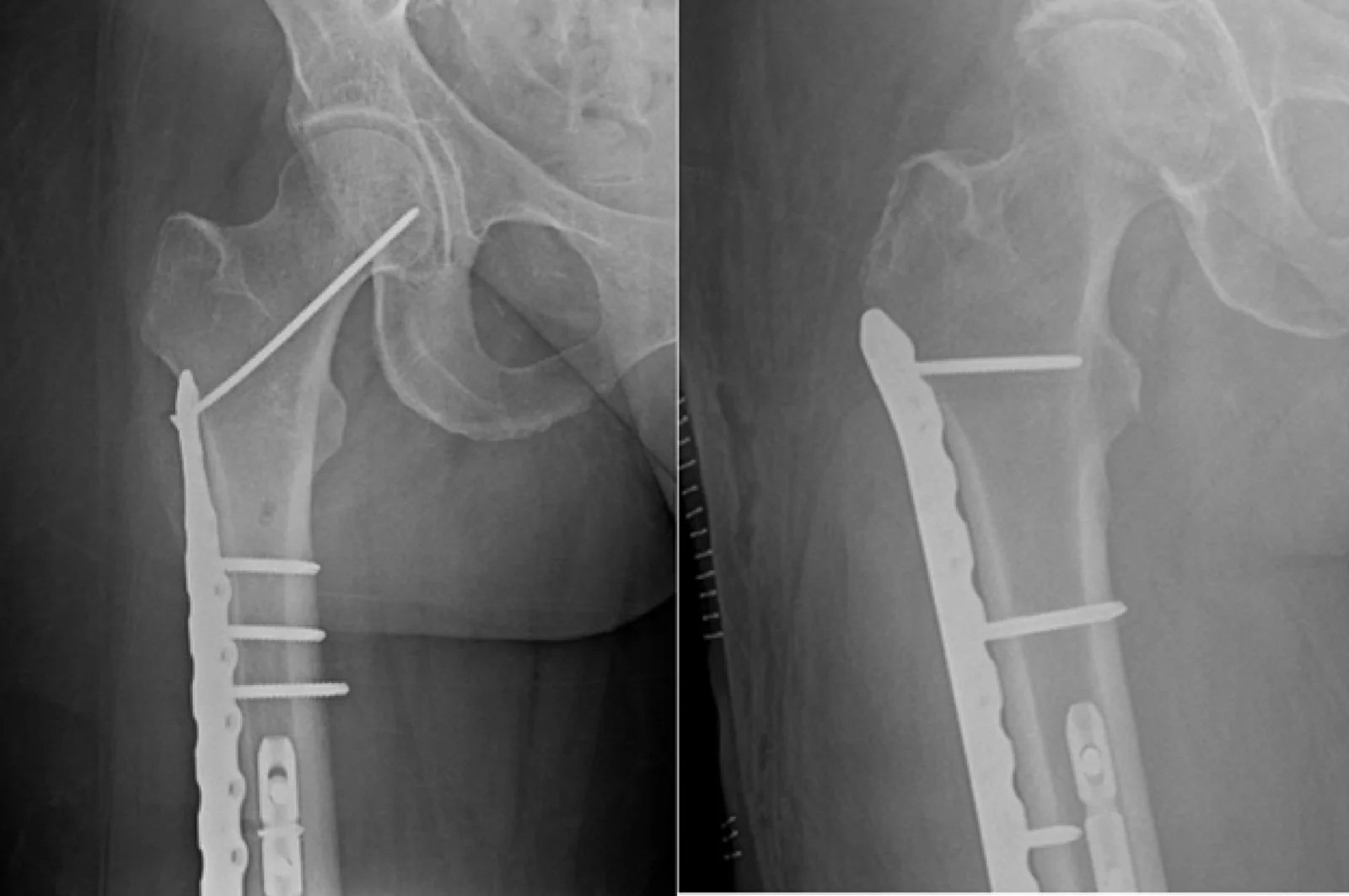
NPC fixation with and without utilization prophylactic femoral neck screw

Baseline demographics, fracture classification, and fixation construct of the cohort were extracted from the medical record (Table 1). Operative notes and patient images in the electronic medical record (EMR) were used to confirm placement of a 4.5 mm cortical prophylactic femoral neck screw (Fig. 1). A patient was determined to have underwent prophylactic fixation if there was either screw insertion into the femoral head through the proximal aspect of a lateral plate or independent screws around the proximal aspect of a retrograde intramedullary nail. Incidence of future hip fracture was determined for each patient based upon record in the EMR of surgical treatment for either an intertrochanteric or femoral neck fracture obtained in a subsequent injury. Data was analyzed using IBM SPSS Statistics Version 27 (IBM Corp., Armonk, NY). Continuous variables were compared using independent samples t-tests, and categorical and binary variables were compared using Chi-square tests. A Kaplan–Meier survival curve was generated for each cohort to compare time to fracture with a log rank test. Significance was determined at *p* < 0.05. Post hoc power analysis was performed using the observed effect sizes, sample sizes, and α = 0.05.

**Table 1:** Cohort demographics

Variable	PS − (n = 232)	PS + (n = 163)	*p*-value
Age, years (mean ± SD)	70.0 ± 11.4	73.7 ± 10.9	0.001
ASA score (mean ± SD)	2.97 ± 0.69	2.88 ± 0.56	0.140
BMI, kg/m^2^ (mean ± SD)	32.6 ± 9.5	31.6 ± 9.3	0.294
*Sex, n (%):*			0.006
Female	159 (68.5%)	132 (81.0%)	
Male	73 (31.5%)	31 (19.0%)	
*Race, n (%):*			0.034
White	172 (74.1%)	143 (87.7%)	
Black/African American	51 (22.0%)	19 (11.7%)	
Asian	2 (0.9%)	0 (0.0%)	
Other/Declined/Unknown	7 (3.0%)	1 (0.6%)	
*Fracture classification, n (%):*			< 0.001
AO 33A	83 (35.8%)	27 (16.6%)	
AO 33B	6 (2.6%)	0 (0.0%)	
AO 33C	57 (24.6%)	30 (18.4%)	
Su 1	37 (15.9%)	17 (10.4%)	
Su 2	37 (15.9%)	63 (38.7%)	
Su 3	12 (5.2%)	26 (16.0%)	
*Diabetes mellitus, n (%):*			0.839
Yes	82 (35.3%)	56 (34.4%)	
No	150 (64.7%)	107 (65.6%)	
*Smoking status, n (%):*			0.025
Current smoker	44 (19.0%)	15 (9.2%)	
Non-smoker	145 (62.5%)	117 (71.8%)	
Never assessed	43 (18.5%)	31 (19.0%)	
*Chronic kidney disease, n (%):*			0.159
Yes	33 (14.3%)	32 (19.6%)	
No	198 (85.7%)	131 (80.4%)	
*Prior total knee arthroplasty, n (%):*			< 0.001
Yes	83 (35.8%)	106 (65.0%)	
No	149 (64.2%)	57 (35.0%)	
*Fixation construct, n (%):*			< 0.001
Nail	97 (42.0%)	3 (1.9%)	
Plate	68 (29.4%)	39 (24.1%)	
Dual plate	5 (2.2%)	8 (4.9%)	
Nail/plate combination	61 (26.4%)	112 (69.1%)	
Median follow-up length, months (IQR)	4.8 (1.8–13.9)	5.8 (3.8–7.7)	0.546

## Results

**Fig. 2:**
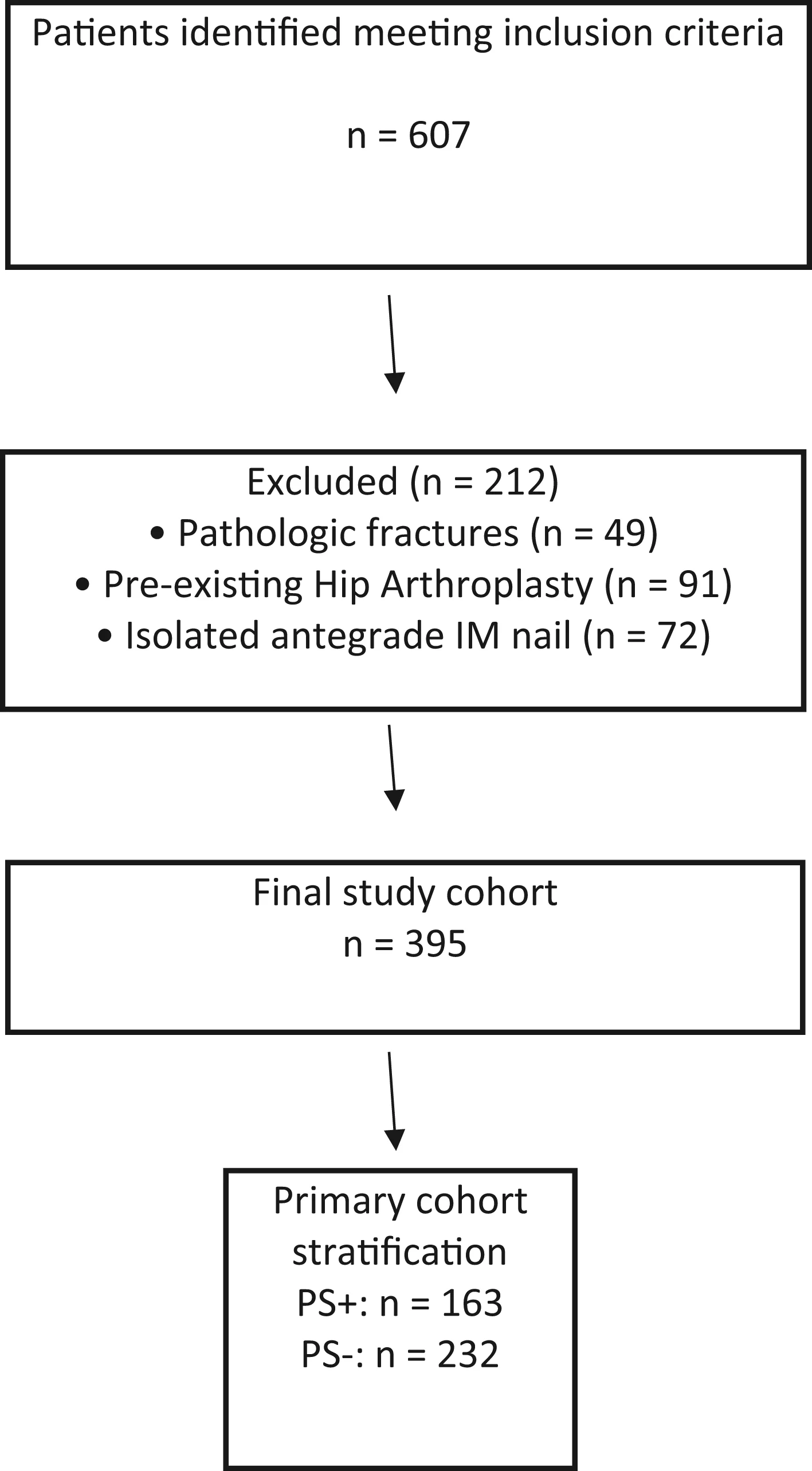
Patient recruitment flowchart

The total cohort of distal femur ORIF patients was 395, with 163 (41%) total patients receiving prophylactic femoral neck screw fixation (PS +) (Fig. 2). Sex distribution differed between the protected and unprotected (PS-) cohorts, with a greater proportion of females in the PS + cohort (n = 132, 81.0% vs. n = 159, 68.5%, *p* = 0.006). The American Society of Anesthesiologists (ASA) score and body mass index (BMI) were statistically similar between groups (*p* = 0.140 and *p* = 0.294). As for fracture classification, the PS- group was mostly represented by AO 33A fracture patterns (n = 83, 35.8%) while the PS + cohort was mostly represented by Su type II (n = 63, 38.7%) (*p* < 0.001). Relevant comorbidities did not show any statistically significant difference. Smoking status showed a significant difference (*p* = 0.025), with fewer current smokers in the PS + group (9.2% vs. 19.0%). There was no significant difference in median follow-up length (4.8 months vs 5.8 months, *p* = 0.546) (Table 1).

The PS + cohort had a higher proportion of periprosthetic fractures than the PS- cohort (65.0% vs. 35.8%, *p* < 0.001). In the PS − cohort, fixation was most frequently achieved with a retrograde nail (n = 97, 42.0%), followed by a lateral plate (n = 68, 29.4%) and a nail-plate combination (n = 61, 26.4%). Only a small proportion underwent dual plating (n = 5, 2.2%). By contrast, the PS + cohort was overwhelmingly treated with a nail-plate construct (n = 112, 69.1%), with fewer cases using a lateral plate (n = 39, 24.1%) or dual plating (n = 8, 4.9%). Use of an isolated retrograde nail was rare in this group (n = 3, 1.9%) (Table 1).

**Fig. 3:**
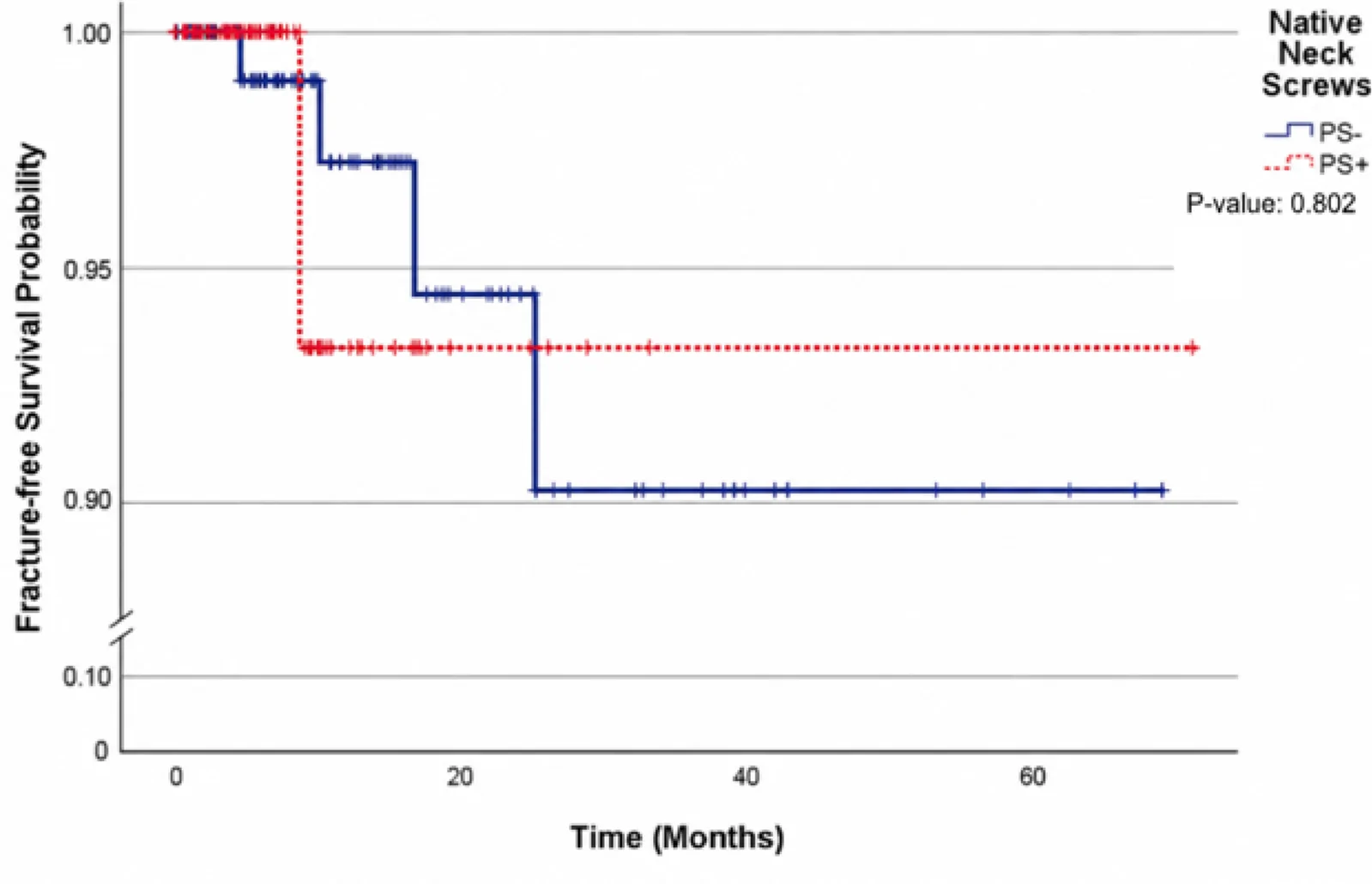
Kaplan–Meier fracture-free survival curve stratified by prophylactic screw status. Tick marks indicate censored observations

In total, there were 6 patients (1.5%) that had documentation of an ipsilateral hip fracture. Of these, 2 had prophylactic screw placement (1.2%, both intertrochanteric fractures) and 4 did not (1.7%, three intertrochanteric fractures and one femoral neck fracture). Of note, all 6 hip fractures were female (Table 2). All characteristics of the hip fracture patients can be found in Table 2. There was no statistically significant difference in femoral neck fracture rate between the PS + and PS − cohorts (*p* = 0.691). Kaplan–Meier analysis also demonstrated no significant difference in fracture-free survival between groups (log-rank *p* = 0.802) (Table 3, Fig. 3). Post hoc power analysis demonstrated that there was an achieved power of only 7.1%.

**Table 2:** Hip fracture demographics

Variable	n (%) or mean ± SD
Age, years (mean ± SD)	79.3 ± 15.8
ASA score (mean ± SD)	3.17 ± 0.98
BMI, kg/m^2^ (mean ± SD)	28.1 ± 11.1
*Sex, n (%):*	
Female	6 (100.0%)
*Race, n (%):*	
Asian	1 (16.7%)
White	5 (83.3%)
*Fracture classification, n (%):*	
AO 33A	1 (16.7%)
AO 33C	1 (16.7%)
Su 2	3 (50.0%)
Su 3	1 (16.7%)
*Diabetes mellitus, n (%):*	
Yes	3 (50.0%)
No	3 (50.0%)
*Smoking status, n (%):*	
Current smoker	1 (16.7%)
Non-smoker	5 (83.3%)
*Chronic kidney disease, n (%):*	
Yes	1 (16.7%)
No	5 (83.3%)
*Prior total knee arthroplasty, n (%):*	
Yes	3 (50.0%)
No	3 (50.0%)
*Ipsilateral lower extremity fracture, n (%):*	
Yes	1 (16.7%)
No	5 (83.3%)
*Fixation construct, n (%):*	
Plate	4 (66.7%)
Nail/plate combination	2 (33.3%)
*Native neck screws, n (%):*	
Yes	2 (33.3%)
No	4 (66.7%)

**Table 3:** Survival curve log rank comparison

Chi-square	df	*p*-value
0.063	1	0.802

## Discussion

This study evaluated prophylactic femoral neck screw placement for prevention of ipsilateral hip fracture and found that fractures still occur despite prophylaxis. Although the overall fracture rate was low and numerically higher in the PS − group, no statistically significant difference was observed. Relevant patient comorbidities such as BMI, chronic kidney disease, and diabetes all did not differ between the two groups, although smoking was more common in the PS − group. It should be noted, however, that both the PS + and PS − cohorts each had almost a fifth of its patients lacking a smoking status, and so this aspect should be assessed with caution. These findings demonstrate that screw placement may not be the sole determinant of future proximal femur fracture risk. For example, construct selection was strongly associated with screw placement, with NPC over-represented in the PS + group. This reflects that screw placement is linked to broader surgical decision-making in establishing stability and thus may indicate that the risk profile for future hip fracture is multifactorial. In other words, stability of the construct as a whole and protection of the entire femur with a long spanning implant or dual construct, in addition to the benefits of early mobility, may be protective in concert as opposed to the independent presence of a screw in the femoral neck. The group receiving prophylactic screw fixation was predominantly comprised of elderly females which is a high-risk group for proximal femur fracture by virtue of osteoporosis. While impossible to say, the fact that the rate of proximal femur fracture was slightly lower in the PS + compared to the PS − (comprised of a higher proportion of males and younger patients), there may still be an underappreciated effect of screw prophylaxis which cannot be completely discounted. Another important consideration is the specificity of the protection that this prophylaxis is providing. Both hip fractures in the PS + group were intertrochanteric fractures, while the only femoral neck fracture observed in this study was in the PS- group. There is a possibility that this prophylaxis is protective against femoral neck fractures but offers minimal protection against other fractures of the hip. Since most of our observed fractures were intertrochanteric, it is impossible to state this with confidence, but larger studies with greater numbers of femoral neck fractures would shed more light on this possibility.

As a whole, the total fracture rate of 1.5% is within the range of reported ipsilateral hip fracture rates after distal femur ORIF of 1–7% [6–8]. Furthermore, there was an ipsilateral hip fracture rate of 1.7% for those without screw placement and 1.2% with screw placement which differs from prior reports of 5% and 0%, respectively [14]. While this specific study lacks the statistical power to make objective claims regarding the efficacy of screw placement, the rate of peri-implant fractures in the prophylactic screw cohort suggests that screw placement alone is not definitively protective. This finding is of importance because it furthers the discussion regarding prophylactic protection of the femur in distal femur fractures. Indeed, the placement of a femoral neck screw could play a role in overall hip fracture prevention but may not be the deciding factor. Other aspects of surgical planning and approach such as specific technique, patient factors, and the fixation construct utilized may amplify or diminish the protective qualities of the femoral neck screw. For example, there were demographic differences between the PS + and PS − cohorts, with the PS + group being significantly more female [14]. Also, although these cohorts did not differ in BMI, there does not seem to be literature investigating its relationship with the protective value of a femoral neck screw in this specific context. As of now, it is unknown as to why these outcomes differ from those of previous analyses, but it is possible that there may be cohort characteristics that are clinically important for fracture susceptibility. Also of note, the PS + group had a higher proportion of periprosthetic distal femur fractures represented as opposed to the PS- group. It has been demonstrated previously that those that undergo total knee arthroplasty (TKA) can be over 50% more likely to sustain a hip fracture within the first 1–3 years after the procedure [16, 17]. While it is unclear from this data how soon the ORIF procedure was after their arthroplasty, it is possible that this increased risk played a role in the decision for screw placement. Indeed, it is possible that risk categorization played a role among cohorts, with periprosthetic fractures deemed as requiring greater protection. The PS + group was also overwhelmingly treated with NPC which reflects the growing adoption of combined fixation strategies in distal femur fracture management. These constructs give increased stability to allow for earlier weight bearing, lower rates of nonunion, and significantly reduced reoperation rates [18–23]. Therefore, the desire for increased fracture stability based on the characteristics of the patient may be related to the decision to employ prophylactic femoral neck fixation.

As seen in these findings, fractures occur even with prophylactic fixation. Biomechanical evidence has suggested that prophylactic femoral neck fixation reduces the incidence of femoral neck fractures under axial loading conditions in the context of intramedullary nailing [12]. However, this does not speak to its efficacy in vivo or in the context of other open reduction internal fixation constructs such as lateral plating or nail-plate combinations. In these two constructs, longer plates can be placed minimally invasively to protect the entire femur, and a screw can be placed in the femoral neck for full protection [14, 24, 25]. In the study institution, prophylactic fixation of the femoral neck has been increasingly utilized, with variable outcomes.

While rare, there are complications that may arise with the use of prophylactic femoral neck screws. Adding another implant to the procedure increases operative time which has been linked to increased complications such as blood loss, urinary tract infection, reoperation, and surgical site infection [26–31]. Also, suboptimal screw placement comes with the risk of generating potential stress risers in osteoporotic bone [32]. Finally, implant strain and screw fracture at the head shaft junction may be possible. Therefore, proper surgical planning and discretion should continue to be employed regarding screw placement, and the risks should be properly weighed against the perceived risk of future hip trauma.

There are notable strengths of this study. First, it includes a large, single-center cohort of 395 patients. Also, the inclusion of both native and periprosthetic distal femur fractures captures a broad patient population that improves the generalizability of the findings. However, there are many limitations to this study. First, as stated before, while this cohort is one of the largest utilized to compare this technique, it may be limited in the power required to make statistically significant comparisons. With the small sample of hip fractures, any multivariable analysis between the cohorts is inconclusive and, therefore, inappropriate. Furthermore, there may be several confounders implicated between cohorts. For example, the PS + cohort was predominantly represented by female patients and periprosthetic femur fractures. Since all of the hip fractures were in female patients, it is unknown to what degree these confounders affect both patient selection for prophylactic screw placement and patient outcomes. However, the presence of fractures in the prophylactic group serves as a call to further investigate prophylactic fixation’s role in hip fracture prevention. Second, this analysis is retrospective and takes place at a single institution, so generalization of findings is limited. Lastly, the use of screw fixation was determined at the surgeon’s discretion, and so there may be unknown confounders determining this selection, such as overall fixation construct and postoperative mobilization protocol. Since all screw placement was treated as equivalent for this study, future directions for this topic should include stratification of results comparing screws inserted through a LLP and those independently inserted around the tip of a rIMN.

## Conclusion

This retrospective study did not identify a significant difference in the rate of subsequent ipsilateral hip fractures between patients who did and did not receive prophylactic femoral neck screw placement during distal femur ORIF. Furthermore, there were documented ipsilateral hip fractures in both groups. While this study lacks the power to directly comment on the treatment effect of prophylactic screw placement, these results suggest that there may be other factors influencing future hip fracture risk in treated distal femur fractures. Surgeon discretion is necessary when assessing when and how to protect the entire femur during these cases in a largely osteoporotic population.

## Data Availability

This study utilized private patient information and is not publically available. Data can be made available upon reasonable request.
